# Combining onabotulinumtoxin A with a CGRP antagonist for chronic migraine prophylaxis: where do we stand?

**DOI:** 10.3389/fpain.2023.1292994

**Published:** 2023-10-27

**Authors:** Lanfranco Pellesi

**Affiliations:** Clinical Pharmacology, Pharmacy and Environmental Medicine, Department of Public Health, University of Southern Denmark, Odense, Denmark

**Keywords:** headache, monoclonal antibodies, pain, real-world evidence, pharmacoeconomics

## 1. Introduction

Chronic migraine involves multiple signaling pathways activated by the release of vasoactive peptides from trigeminovascular neurons (1). For migraine prevention, onabotulinumtoxin A and calcitonin gene-related peptide (CGRP) antagonists are effective and well tolerated therapies. Onabotulinumtoxin A injections are supposed to attenuate the pain by preventing neuropeptide release and downregulating nociceptive ion channels in specific head and neck regions characterized by the presence of sensory nerve endings (2–7). Directly inhibiting the CGRP pathway is another targeted approach for migraine prevention (8). Four anti-CGRP monoclonal antibodies (anti-CGRP mAbs) are approved in different countries, including three antibodies directed against the CGRP peptide (eptinezumab, fremanezumab and galcanezumab) and one binding to the CGRP receptor (erenumab). More recently, small-molecule CGRP receptor antagonists (or gepants) have been authorized for similar purposes.

Migraine prevention in patients who experience multiple therapy failures is challenging. Combining onabotulinumtoxin A with a single CGRP antagonist may be indicated in patients who continue to experience migraine pain despite receiving either therapy. Preclinical studies showed that the clinical benefits of combined therapy may be additive or synergistic in nature (9). In animals, onabotulinumtoxin A mainly prevents activation of unmyelinated C-fibers and consequent CGRP release (10), whereas fremanezumab inhibits activation of CGRP receptors on thinly A*δ*-fibers (11). Despite the European recommendations are to discontinue onabotulinumtoxin A before initiation of an anti-CGRP mAb (12), the American Headache Society consensus statement recognizes the value of combining preventatives from different drug classes in patients with a suboptimal response (13).

This Opinion summarizes the real-world knowledge on combining onabotulinumtoxin A with a single CGRP antagonist in chronic migraine patients. The intention is to interpret published real-world data to offer an updated clinical perspective and opportunities for future research.

## 2. Real-world studies

Table 1 summarizes the main results from published literature. In 2020, an observational prospective study showed the effects of onabotulinumtoxin A combined with erenumab in 45 chronic migraine patients that previously failed more than three preventive measures (14). Combining onabotulinumtoxin A with erenumab was more effective in reducing monthly migraine days than combining erenumab with an oral prophylactic (*n* = 11) and/or erenumab alone (*n* = 13). A retrospective study described the real-world administration of onabotulinumtoxin A with erenumab (either 70 or 140 mg) for 6 months in 10 chronic migraine patients (15). Another retrospective case series evaluated the effects of administering either fremanezumab, galcanezumab or erenumab in 36 chronic migraine patients who received at least 2 cycles of onabotulinumtoxin A (16). Half of the patients reported an improvement in their headache burden after the addition of the anti-CGRP mAb.

**Table 1:** Summary of findings concerning the dual therapy with onabotulinumtoxin A and a CGRP antagonist.

Study	Year	Study type	Efficacy results
Boudreau GP (14)	2020	Real world, prospective, observational study	Dual treatment with erenumab and onabotulinumtoxin A reduced monthly migraine days in 45 CM patients (65%)
Robblee et al. (15)	2020	Retrospective, observational study	The number of monthly headache days was reduced by a mean of 2.5 days at 6 months in 10 patients receiving onabotulinumtoxinA and erenumab
Ozudogru et al. (16)	2020	Case series	Half of the patients (18/36) demonstrated an improvement in their headache burden after the addition of an anti-CGRP mAb
Blumenfeld et al. (17)	2021	Retrospective chart review	Compared with onabotulinumtoxinA alone, adding an anti-CGRP mAb provided meaningful reductions in monthly headache days up to one year of dual therapy
Toni et al. (18)	2021	Case series	After the addition of an anti-CGRP mAb, monthly headache days and headache severity were further reduced in 17 CM patients
Cohen et al. (19)	2021	Retrospective chart review	After the addition of an anti-CGRP mAb, CM patients experienced a further decrease of 5.7 monthly headache days
Armanious et al. (20)	2021	Retrospective chart review	CM patients treated with erenumab in addition to onabotulinumtoxin A injections reported a further reduction of monthly migraine days and monthly headache days
Alpuente et al. (21)	2021	Real world, prospective, observational study	No significant differences were found in clinical parameters between CM patients using an anti-CGRP mAb (*n* = 33) and CM patients under dual therapy (*n* = 12)
Silvestro et al. (22)	2021	Case series	Combining onabotulinumtoxin A with erenumab further reduced monthly headache days, headache severity, symptomatic drug intake and disability in ten refractory CM patients
Mechtler et al. (23)	2022	Retrospective, longitudinal study	After 12 months of combination treatment with onabotulinumtoxin A and an anti-CGRP mAb, monthly headache days were reduced by 4.6 days/month from baseline
Argyriou et al. (24)	2022	Multicenter, retrospective chart review	Dual therapy was effective and was associated with clinically meaningful improvement in efficacy variables in 14 out of 19 refractory CM patients
Nandyala et al. (25)	2022	Retrospective, cohort study	Combining erenumab with onabotulinumtoxin A reduced monthly migraine days and monthly headache days in 50 CM patients

CM, chronic migraine.

In 2021, a retrospective study evaluated the effects of ≥2 consecutives cycles of onabotulinumtoxin A and ≥1 month of subsequent combination treatment with an anti-CGRP mAb in 257 adults with chronic migraine (17). Prescribed mAbs were erenumab (78%), fremanezumab (6%), and galcanezumab (16%). Compared with onabotulinumtoxin A alone, mean monthly headache days decreased significantly at 6, 9, and 12 months after initiation of dual treatment. The combined therapy was well tolerated, with the most common adverse event being constipation (8.6%). A case series described the effects of combining onabotulinumtoxin A with an anti-CGRP mAb in 17 chronic migraine patients who had a partial or poor response to onabotulinumtoxin A (18). Among them, 9 started fremanezumab, 4 started erenumab and 4 initiated galcanezumab. Eleven patients improved, 4 patients experienced no improvement and 2 patients reported a worsened headache severity. No significant adverse events were described. A retrospective study reviewed medical records of 153 chronic migraine patients receiving onabotulinumtoxin A who were subsequently prescribed an anti-CGRP mAb 19). Patients received erenumab (58%), fremanezumab (9%) or galcanezumab (33%). The addition of an anti-CGRP mAb was safe and further decreased the monthly headache days. A retrospective study investigated the effects of adding erenumab (either 70 mg or 140 mg) in 78 chronic migraine patients receiving onabotulinumtoxin A (20). The vast majority of patients failed at least three preventive therapies. The dual therapy further reduced monthly headache days and monthly migraine days up to 90 days after the beginning of the combined treatment. A prospective observational study evaluated chronic migraine patients who were partial or nonresponders to onabotulinumtoxin A and initiated a treatment with either erenumab or galcanezumab (21). After three months, no significant differences were found between an anti-CGRP mAb monotherapy (*n* = 33) and dual therapy with onabotulinumtoxin A (*n* = 12) in terms of monthly headache days and monthly migraine days. A case series reported significant benefits in 10 chronic migraine patients receiving onabotulinumtoxin A combined with erenumab (140 mg), compared with either therapy alone (22). The dual therapy resulted in a significant reduction of symptomatic drug intake and migraine-related disability.

In 2022, a retrospective single-center study evaluated 148 chronic migraine patients treated with ≥2 consecutive cycles of onabotulinumtoxin A before ≥1 month of combination treatment with either erenumab, fremanezumab or galcanezumab (23). Erenumab was prescribed to 56.7% of patients, fremanezumab to 42.6% and galcanezumab to 0.7%. After adding an anti-CGRP mAb, monthly headache days were further reduced up to 12 months of continuous dual therapy. Adverse events were reported by 18 patients (12.2%), with the most common being constipation and injection-site reactions. A retrospective study evaluated the medical records of 19 treatment-refractory chronic migraine patients who failed two oral migraine prophylactics, at least three consecutive onabotulinumtoxin A cycles and at least three consecutive sessions with either fremanezumab or erenumab (24). Patients were eventually switched to dual therapy with onabotulinumtoxin A and any of the already-given anti-CGRP mAbs. Compared either to baseline or at discontinuation of each monotherapy intervention, meaningful improvements were observed in headache frequency, disability and quality of life in the majority of patients after at least two courses of dual therapy. A retrospective case series evaluated the effects of combining onabotulinumtoxin A and erenumab in 50 chronic migraine patients receiving onabotulinumtoxin A, who were additionally started on monthly erenumab (either 70 mg or 140 mg) (25). Compared to onabotulinumtoxin A alone, patients experienced a reduction in monthly migraine and headache days without relevant side effects.

## 3. Discussion

The introduction of CGRP antagonists in the migraine armamentarium constitutes a targeted approach for achieving prophylaxis. Nonetheless, focusing on a specific mechanism of action rarely achieves migraine freedom. The percentage of patients using anti-CGRP mAbs with ≥50% reduction in monthly migraine days is approximately 50% (26, 27). Thus, dual targeting with onabotulinumtoxin A and a CGRP antagonist deserve consideration in individuals with sustained migraine and disability. Combining onabotulinumtoxin A with an anti-CGRP mAb has been reported only in real-world studies, therefore prone to the placebo effect. In addition, the relative contribution of onabotulinumtoxinA and antibodies on migraine prevention could not be adequately disentangled. Regarding the combination of onabotulinumtoxin A with gepants, a single real-world study evaluated the effectiveness of ubrogepant (50 or 100 mg) for the acute treatment of migraine in onabotulinumtoxin A users (28). After one month, satisfaction with ubrogepant in combination with onabotulinumtoxin A was reported by 69.8% of participants. In a phase 2b/3 trial, concomitant treatment with rimegepant and onabotulinumtoxin A was allowed if the latter was used at a stable dose and frequency (29). A *post-hoc* analysis of the trial may be relevant to further elucidate the clinical course of the dual treatment.

Real world data have several limitations, including the retrospective study design, lack of a randomization process, missing control group and heterogeneous outcomes. Only a single study evaluated the combination of erenumab with onabotulinumtoxin A in a prospective way, whereas the design of the other studies included in this Opinion was retrospective (14). The majority of studies showed that partial responders to onabotulinumtoxin A may obtain incremental headache relief by adding an anti-CGRP mAb. The dual treatment was considered safe, with the most common side effects being constipation (often associated with the erenumab administration) and injection-site reactions. A pooled analysis found that combining onabotulinumtoxin A with an anti-CGRP mAb provides a mean reduction of almost 3 monthly headache days after three months of treatment (30). The addition of an anti-CGRP mAb may be particularly useful in migraine patients experiencing a “wear-off” of onabotulinumtoxin A effect, providing an additional benefit in those patients experiencing an incomplete response to onabotulinumtoxin A alone (16, 19). Further sufficiently powered, placebo-controlled studies are warranted to shed light on potential additive or synergistic effects of combining onabotulinumtoxin A with a CGRP antagonist.

Dual therapies are common in migraine, including combinations of oral treatments or combinations of an oral with an injectable treatment (31). However, combining onabotulinumtoxin A with a CGRP antagonist remains challenging regardless clinical judgment. Onabotulinumtoxin A, anti-CGRP mAbs and gepants are highly expensive therapies that patients wishing to combine them have to fully cover the cost of at least an entire medication. Several national systems do not grant reimbursement for combining onabotulinumtoxin A with a CGRP antagonist. In these uncertain times, chronic migraine patients may be reluctant to pay a significant amount of money for receiving such dual therapy. In the aforementioned real-world studies, one of the most common reasons for discontinuing either therapy was lack of insurance reimbursement (17, 23). When the coverage for concurrent treatment with onabotulinumtoxin A and an anti-CGRP mAb was denied, five patients reported an increased headache burden after discontinuing the anti-CGRP mAb (19). Pharmacoeconomic evaluations in adults with chronic migraine support the cost-effectiveness of onabotulinumtoxin A and anti-CGRP mAbs, but whether their co-administration is cost-effective is unknown (32). New health economy studies will assess the cost-benefit ratio of this dual therapy, potentially justifying a more aggressive approach for the treatment of refractory chronic migraine.
